# Absorption of Water by Plants

**Published:** 1883-05

**Authors:** 


Absorption of Water by Plants.—An enormous quantity of water passes through the roots of plants. An English experimenter has ascertained that for every pound of mineral matter assimilated by a plant, an average of 2,000 pounds of water is absorbed. At the French Agricultural Observatory of Montsouris it was found that in rich soil, 727 pounds of water passed through the roots of wheat plants for every pound of grain produced; while in a very poor soil, 2693 pounds passed through the wheat roots for every pound of grain.

